# Special Issue: “Molecular Background of Obesity and Its Impact on Therapeutic Strategies”

**DOI:** 10.3390/ijms27010040

**Published:** 2025-12-19

**Authors:** Lukasz Buldak

**Affiliations:** Department of Internal Medicine and Clinical Pharmacology, Medical University of Silesia, Medykow 18, 40-752 Katowice, Poland; lbuldak@gmail.com

## 1. Introduction

The global burden of obesity has forced a paradigm shift in biomedical research, moving beyond the historical view of simple caloric imbalance to recognizing obesity as a complex, systemic pathology governed by intricate molecular signaling networks [1]. Today, obesity is considered a chronic disease associated with chronic low-grade inflammation, dysregulated hormonal axes and profound alterations in cellular energy metabolism, which lead to secondary comorbidities, including type 2 diabetes mellitus (T2DM), metabolic dysfunction-associated steatotic liver disease (MASLD), cardiovascular disease and malignancy [2].

This Special Issue of the International Journal of Molecular Sciences, entitled “Molecular Background of Obesity and Its Impact on Therapeutic Strategies,” compiles a collection of high-quality reviews and original research articles. These contributions address the molecular mechanisms associated with obesity, ranging from fetal epigenetic programming to adult inflammatory signaling, and evaluate how these findings are being translated into novel pharmacological and nutraceutical interventions.

## 2. The Inflammatory and Hormonal Factors Related to Obesity

A central theme emerging from this Special Issue is the concept of “metaflammation”—a sterile, chronic inflammatory state. In their comprehensive review, Campos-Bayardo et al. (Contribution 1) focus on the role of Toll-like receptors (TLRs) in bridging innate immunity and metabolic dysfunction. While they are evolutionarily designed to detect pathogens, TLRs (particularly TLR2 and TLR4) are activated by saturated fatty acids in obesity. This activation triggers downstream cascades, including NF-κB, which upregulate proinflammatory cytokines that directly impair insulin signaling. The authors detail how this TLR-driven inflammation propagates from adipose tissue to the liver and vasculature, accelerating the progression of NAFLD and atherosclerosis.

The perspective is supported by Szydlowska-Gladysz et al. (Contribution 2), who explore the endocrine paradox of the insulin-like growth factor (IGF) axis. Their systematic review highlights a critical duality: while IGF-1 and IGF-2 are essential for pediatric growth, their chronic elevation in obese people poses significant long-term risks due to hyperinsulinemia. The authors discuss how the IGF axis promotes accelerated prepubertal growth and bone maturation while simultaneously fueling neoplastic transformation, linking obesity directly to increased cancer risk. This shows the need to view obesity not just as a metabolic disorder but as a multi-system proliferative disease.

## 3. The Epigenetic Concept

The molecular roots of obesity often precede birth. The “Developmental Origins of Health and Disease” (DOHaD) hypothesis is explored in an original study by Mas-Parés et al. (Contribution 3). Using a swine model, highly translational to human physiology, the authors demonstrated that gestational caloric restriction significantly altered the metabolic features of the offspring. Piglets born to underfed sows exhibited a “pre-metabolic syndrome” profile, characterized by dyslipidemia and inflammation, despite normal birth weights.

Interestingly, this study attributed these phenotypic changes to specific alterations in the DNA methylome of visceral adipose tissue. Genes regulating lipogenesis (*FASN*) and insulin signaling (*PRKCZ*) were hypermethylated, providing a molecular mechanism for the observed metabolic inflexibility. The authors also explored early postnatal treatment with metformin, which might reverse adverse epigenetic programming.

## 4. Incretins and the Gut–Liver Axis

As our understanding of pathophysiology deepens, so does our therapeutic arsenal. Nicze et al. (Contribution 4) provide an extensive review of current and future anti-obesity strategies, categorizing them by their molecular targets: central appetite control, gastrointestinal motility and energy expenditure. The review highlights the revolutionary impact of incretin-based therapies, such as GLP-1 and GIP receptor agonists, which have surpassed their initial indication in diabetes to become the gold standard for weight management.

The mechanistic depth of these drugs is further explored in an original research article by Soto-Catalán et al. (Contribution 5). Investigating the effects of semaglutide in leptin-receptor-deficient (*db/db*) mice, the authors revealed that the GLP-1 analog’s benefits extended beyond weight loss. Semaglutide treatment significantly ameliorated hepatic steatosis and inflammation. Notably, this hepatoprotection occurred without a significant reduction in food intake in this hyperphagic model, suggesting direct hepatic effects. Molecular analysis showed a downregulation of de novo lipogenesis markers (specifically *Scd1*) and a remodeling of the hepatic lipidome toward beneficial polyunsaturated fatty acids. This supports the positioning of GLP-1 receptor agonists as frontline therapies for MASLD.

## 5. Microbiome and Obesity

Finally, this Special Issue addresses the potential of functional foods to modulate metabolic signaling. Maruta et al. (Contribution 6) presents original research on fermented milk enriched with specific *Bifidobacterium* strains (e.g., Bif-15). In a rat model of obesity, this probiotic intervention suppressed weight gain and reduced visceral fat. The study elucidates a specific mechanism: *Bifidobacterium*-derived acetate acts as a signaling molecule, activating AMPK in the liver to suppress lipogenesis and stimulating GPR43 in skeletal muscle to enhance mitochondrial biogenesis. This highlights the gut–muscle and gut–liver axes as viable targets for non-pharmacological interventions.

## 6. Conclusions

The contributions to this Special Issue illustrate that obesity is a disease of dysregulated signaling nodes—involving crosstalk between the genome, the immune system and the microbiome. The transition from the simple “calories in, calories out” model to a molecular-based understanding is paving the way for precision medicine. By combining advanced pharmacotherapies such as semaglutide with targeted nutritional strategies and early-life interventions, we can develop a comprehensive approach to halting the metabolic pandemic.

## Data Availability

Not applicable.
